# Identification of CMY-190, a novel chromosomally encoded AmpC β-lactamase, and plasmid-encoded KPC-2 in a clinical isolate of *Citrobacter youngae*

**DOI:** 10.3389/fmicb.2025.1526882

**Published:** 2025-02-17

**Authors:** Zeshi Liu, Siquan Shen, Xue Zhang, Jing Lei, Chengkang Tang, Shi Wu, Ke Lei, Jian Yin, Yanping Zhang, Yan Guo, Yan Geng, Fupin Hu

**Affiliations:** ^1^Institute of Antibiotics, Huashan Hospital, Fudan University, Shanghai, China; ^2^Department of Clinical Laboratory, The Second Affiliated Hospital of Xi’an Jiaotong University, Xi’an, China; ^3^Key Laboratory of Clinical Pharmacology of Antibiotics, Ministry of Health, Shanghai, China

**Keywords:** *Citrobacter youngae*, *bla*_CMY-190_, AmpC β-lactamase, *bla*_KPC-2_, carbapenemase-producing *Enterobacterales*

## Abstract

This study investigates the antibiotic resistance phenotype and genotype of *Citrobacter youngae* strain YS01, isolated from a peritoneal effusion sample, focusing on both chromosomal and plasmid-mediated resistance mechanisms to inform clinical antibiotic therapy. Our results reveal the presence of the chromosomally encoded β-lactamase CMY-190 and the plasmid-encoded carbapenemase KPC-2, which confer resistance to cephalosporins and carbapenems, respectively. CMY-190 exhibits substrate and inhibition profiles similar to AmpC β-lactamases and shares 88.05% amino acid identity with the plasmid-encoded enzyme CFE-2 from *Citrobacter freundii* pJA99. DNA sequence analysis identified the *ampR* gene upstream of both *bla*_CMY-190_ and *bla*_KPC-2_. In addition, genes identified surrounding the *ampR*–*ampC* regions in *C. youngae*, including *ORF1*, the fumarate operon (*frdABCD*), *blc*, and *lolB*, a DNA fragment not present in other *Citrobacter* species. The *ampR*–*ampC* genes were cloned into the PHSG398 vector and expressed in *Escherichia coli* DH5α, with the transformed strain showing partial resistance to cephalosporins. The *bla*_KPC-2_ was carried by Tn*1721*, previously identified mainly in Asian strains of *Klebsiella pneumoniae*. The expression of KPC-2 was confirmed by the conjugation of the donor bacterium *C. youngae* with *E. coli* J53 and by the transformation of the plasmid containing *bla*_KPC-2_ into *E. coli* DH5α, with all the transformed strains demonstrating resistance to carbapenems and elevated carbapenem MICs. To the best of our knowledge, this is the first report of a novel chromosomally encoded AmpC β-lactamase gene, *bla*_CMY-190_, and the emergence of *bla*_KPC-2_ in *C. youngae*.

## Introduction

Infections caused by carbapenemase-producing *Enterobacterales* are on the rise and represent a significant threat to global healthcare services (Zhang et al., 2017). Among the various types of carbapenemases, KPC and NDM are the two most prominent classes found predominantly in Enterobacterales (Yang et al., 2023). Patients with CPE infections often face limited treatment options due to the scarcity of effective antibiotics (Grundmann et al., 2017). Historically, the primary clinical burden of CPE has been the increasing incidence of hospital-acquired infections caused by *Klebsiella pneumoniae* and *Escherichia coli*. However, other carbapenemase-producing *Enterobacterales* species, including *Enterobacter cloacae* complex, *Serratia marcescens*, and *Citrobacter youngae*, have also been identified in hospital settings (Nordmann and Poirel, 2014). Recent studies have highlighted the emergence of carbapenem-resistant *Citrobacter* species in various sites, such as the human gut, respiratory tract, urinary tract, and bloodstream, associated with severe diseases such as neonatal meningitis and brain abscesses (Yang et al., 2018; Fupin et al., 2022; Bitar et al., 2019). *C. youngae*, a Gram-negative member of the *Enterobacterales*, was originally isolated from human faces and was long considered a rare nosocomial pathogen. A 12-year study revealed that *C. freundii* and *C. koseri* are the two most commonly isolated species in clinical *Citrobacter* infections, with infections caused by other species, including *C. youngae*, accounting for only 5% of cases. The overuse and misuse of antibiotics have contributed to an increase in bacterial resistance and made treatment more difficult. In addition, concerns have been raised about food safety and cross-infection in hospital settings (Lee et al., 2019). *C. youngae* infections have been reported in neonates, young children, and immunocompromised patients, resulting in conditions such as bloodstream, urinary tract, gastrointestinal tract, and abdominal cavity infections (Zhang et al., 2008; Song et al., 2023; Al-Mulla et al., 2014; Chen et al., 2013; McAteer et al., 2023).

In *C. youngae*, the chromosomally encoded AmpC β-lactamase (cAmpC) plays a key role in mediating resistance to β-lactam antibiotics. This enzyme can hydrolyse a wide range of β-lactams, including cephalothin and extended-spectrum cephalosporins, and is remarkably unaffected by traditional β-lactamase inhibitors (Ranjan and Ranjan, 2013). Overexpression of chromosomal AmpC β-lactamase, coupled with reduced outer membrane permeability, often results in decreased susceptibility to carbapenems, particularly imipenem. As a result, clinical microbiology laboratories may have difficulty in accurately diagnosing carbapenemase production in these species (Woodford et al., 2007). Epidemiological studies have shown that *bla*_CMY_ genes encoding AmpC β-lactamases are widely distributed in Enterobacterales. These genes are often associated with multidrug resistance and have been detected in both plasmid-encoded and chromosomally encoded forms (Lee et al., 2019). The *bla*_CMY-2_ is one of the most commonly reported AmpC β-lactamase genes in Enterobacterales, including *Escherichia coli*, *Salmonella* spp., and *Klebsiella pneumoniae* (Zhang et al., 2008; Song et al., 2023; Al-Mulla et al., 2014; Chen et al., 2013; McAteer et al., 2023). Their global spread poses a significant challenge to antimicrobial therapy, particularly in hospital-acquired infections (Ranjan and Ranjan, 2013).

According to previous epidemiological studies, OXA-48-like carbapenemases are the most commonly found in *Citrobacter* species, followed by NDM, VIM, and others (Biez et al., 2023). While KPC-type carbapenemases are highly prevalent in carbapenem-resistant *Enterobacterales* (CRE) overall, they are less commonly detected in *Citrobacter* species. Research has shown that *Citrobacter* spp. harbor a wide variety of plasmids, including a relatively high proportion of carbapenemase-encoding plasmids, suggesting that plasmid-mediated transfer of carbapenemase genes can occur between *Citrobacter* spp. and other bacteria (Arana et al., 2017). The emergence of the *bla*_KPC_ gene in *C. youngae* poses a serious public health threat. Our study identifies the presence of *bla*_KPC_ and the novel *bla*_CMY_ variant *bla*_CMY-190_ (accession number OR896917) in *Citrobacter* spp. and highlights the importance of continuous monitoring and vigilance for this emerging resistance mechanism.

## Materials and methods

### Strain and antimicrobial susceptibility test

A carbapenem-resistant *C. youngae* strain was isolated from the peritoneal effusion at a tertiary hospital in Shaanxi Province, Northwest China. Strain identification was conducted using Illumina MiSeq technology, which is capable of generating millions of short (100–300 bp), low-error (0.1%) paired-end reads. Minimum inhibitory concentrations (MICs) were determined using the Clinical and Laboratory Standards Institute (CLSI) broth microdilution method. With the exception of tigecycline, colistin, and cefoperazone–sulbactam, all drug breakpoints were interpreted according to CLSI M100-33 guidelines (Clinical and Laboratory Standards Institute, 2023). Tigecycline MICs were interpreted using the US Food and Drug Administration (FDA) *Enterobacterales* breakpoint (FDA, 2021), and the colistin MICs were interpreted using the European Committee on Antimicrobial Susceptibility Testing (EUCAST) standards. Quality control strains, *E. coli* ATCC 25922 and *Pseudomonas aeruginosa* ATCC 27853, were used for antimicrobial susceptibility testing. Carbapenemase phenotypes were detected using the imipenem-EDTA double-disk synergy test and the NG-Test Carba-5, while carbapenemase genes (KPC, NDM, OXA, IMP, and VIM) were confirmed using the PCR.

### Conjugation, transformation, and plasmid sequencing

Conjugation and transformation experiments were carried out to investigate plasmid transfer. Briefly, *C. youngae*, a *bla*_KPC-2_-positive isolate, was used as a donor strain, while *E. coli* J53 (azide-resistant) and *E. coli* DH5α were used as recipient strains. The conjugation test was carried out on Mueller–Hinton (MH) agar containing azide (100 μg/mL) and ampicillin (50 μg/mL), while the transformation test was performed on MH agar with ampicillin (50 μg/mL). The presence of the *bla*_KPC-2_ gene and other resistance genes essential for conjugation was confirmed using antimicrobial susceptibility testing, PCR, and DNA sequencing. Identification of each conjugate colony was performed using matrix-assisted laser desorption/ionization time-of-flight mass spectrometry (MALDI-TOF MS) with MALDI Biotyper software (bioMerieux, located in Marcy-l’Etoile, France) and further confirmed by next-generation sequencing (NGS). Plasmids carrying the *bla*_KPC-2_ gene from the conjugants were extracted using the Qiagen Midi Kit (Qiagen, Hilden, Germany) and sequenced on the Illumina NovaSeq (Illumina, San Diego, CA, United States) short-read sequencer (150-bp paired-end reads). Sequencing reads were trimmed using a sickle (GitHub) and assembled *de novo* using SPAdes 3.12.0. Baseline calibration and assembly evaluation were done using Pilon 1.18. Open reading frame prediction and annotation were performed using RAST version 2.02 and BLAST at NCBI. Plasmid replicon types were determined using a PCR-based plasmid replicon typing method (Carattoli et al., 2005). Plasmid comparisons were performed using BRIG (Alikhan et al., 2011).

### Cloning experiments and induced experiments

The *bla*_CMY-190_ gene and its upstream promoter region were amplified from *C. youngae*-YS01. The pHSG398 vector was digested with *Eco*RI and *Kpn*I using the ClonExpress RII One-Step Cloning Kit (Vazyme Biotech Co., Ltd., Nanjing, China). The linearised vector, purified PCR product, buffer, and enhanced recombinase were mixed and incubated for 20 min to obtain the recombinant vector. This recombinant vector was then introduced into *E. coli* DH5α by chemical transformation, followed by the determination of minimum inhibitory concentrations (MICs). In the presence of β-lactamase inducer (cefoxitin 10 μg/mL or cefotaxime 8 μg/mL), *E. coli* DH5α-PHSG398 and the positive control *S. marcescens* were incubated at 37°C for 2 h. The β-lactamase activity was then determined by a spectrophotometric method using the Amplite Beta-Lactamase Activity Assay Kit (AAT Bioquest Inc., America). A chromogenic β-lactam substrate (cephalosporin) was used, which changes color from yellow to red upon hydrolysis by β-lactamase. The assay was performed using an absorbance microplate reader, measuring the OD ratio at wavelengths of 490 nm to 380 nm.

### Whole-genome sequencing and bioinformatic analysis

Genomic DNA was extracted from overnight cultures of single bacterial colonies using a genome extraction kit according to the manufacturer’s instructions (Vazyme, China). DNA was sequenced using Illumina short-read sequencing (150 bp paired-end reads) (Illumina, San Diego, CA, United States). *De novo* sequence assembly was performed using SPAdes 3.12.0. For isolates suspected of carrying plasmid-encoded carbapenemases, long-read sequencing was conducted using the MinION instrument (Oxford Nanopore Technologies, Oxford, United Kingdom). Long-read sequencing libraries were prepared and multiplexed using a rapid multiplex barcoding kit (Oxford Nanopore Technologies catalog number SQK-RBK004) and sequenced on R9.4.1 flow cells. Base calling of raw reads was performed using Guppy v2.3.1 (Oxford Nanopore Technologies, Oxford, United Kingdom), and hybrid assembly, incorporating both Illumina short reads and Oxford Nanopore long reads, was performed using Unicycler v0.4.8-beta (Wick et al., 2017). Antimicrobial resistance gene analysis and draft genome annotation were performed using BacWGSTdb. Multilocus sequence typing (MLST) was performed using the mlst tool.^1^ Genomic comparison of closely related isolates was performed using Proksee.^2^ Antimicrobial resistance genes and plasmids were identified using the BLASTn analysis of assembled contigs against the ResFinder and PlasmidFinder databases, using a cutoff of 80% sequence identity and 80% sequence coverage. A maximum likelihood phylogenetic tree was reconstructed using MEGA11.

## Results

### Overview of the *Citrobacter youngae* isolate

*Citrobacter youngae* was recovered from the peritoneal effusion of a 62-year-old female patient hospitalized for acute liver failure. During her hospital stay, the patient developed a fever, marked abdominal distension, weakness, malaise, and chest tightness on exertion. Initial treatment included intravenous administration of meropenem (1 g Q8H) for 4 days. The patient had several comorbidities, including hepatic malignancy, decompensated cirrhosis secondary to hepatitis B, ascites, acute renal failure, uremia, and portal vein thrombosis. Subsequently, a KPC-2 enzyme-producing *C. youngae* was isolated from the ascitic fluid, and the intravenous therapy was changed to ceftazidime–avibactam (2.5 g Q8H) for 7 days. Following this treatment, the patient’s temperature normalized, and the infection was effectively controlled. She was discharged 21 days after admission. The antimicrobial susceptibility profile of the *C. youngae* isolate is detailed in Table 1. The isolate was susceptible to ceftazidime–avibactam (MIC = 1 μg/mL), amikacin (MIC = 2 μg/mL), tigecycline (MIC = 0.125 μg/mL), and colistin (MIC≤0.25 μg/mL). In contrast, it was resistant to imipenem (MIC = 8 μg/mL), meropenem (MIC = 16 μg/mL), cefepime (MIC≥32 μg/mL), cefoperazone–sulbactam (MIC = 64 μg/mL), aztreonam (MIC≥128 μg/mL), and ciprofloxacin (MIC = 8 μg/mL). The results of ATCC strains in this study were within the quality control range.

**Table 1:** Susceptibility of *C. youngae* clinical isolate, conjugant, and recipient to antimicrobial agents.

Strain	β-lactamase	MIC (μg/mL)														
		IPM	MEM	MEV	CZA	AMK	FEP	CAZ	CRO	ATM	CIP	SCF	TZP	TGC	COL	SXT
*C. youngae*-YS01	KPC-2, CTX-M-3	8	16	0.06	1	2	32	32	32	128	8	64	256	0.125	0.25	16
*C. youngae*-YS01-*E. coli* J53	KPC-2, CTX-M-3	1	2	0.03	0.125	2	8	16	32	128	0.25	64	128	0.125	0.25	2
*C. youngae*-YS01-*E. coli* DH5α	KPC-2, CTX-M-3	2	4	0.03	0.125	2	8	16	32	128	0.5	64	256	0.125	0.25	2
*E. coli* DH5α	-	0.125	0.03	0.03	0.125	2	0.25	0.25	0.25	1	0.06	1	4	0.125	0.25	0.25
*E. coli* J53	-	0.25	0.03	0.03	0.125	2	0.25	0.5	0.25	1	0.06	1	4	0.125	0.25	0.25

IPM, imipenem; MEM, meropenem; MEV, meropenem–vaborbactam; CZA, ceftazidime–avibactam; AMK, amikacin; FEP, cefepime; CAZ, ceftazidime; CRO, ceftriaxone; ATM, aztreonam; CIP, ciprofloxacin; SCF, cefoperazone–sulbactam; TZP, piperacillin–tazobactam; TGC, tigecycline; COL, colistin; SXT, trimethoprim–sulfamethoxazole.

### Conjugation and transformation experiment

The study also identified the presence of the plasmid-encoded *bla*_KPC-2_ gene in *C. youngae*, marking the first detection of this gene within this bacterial species. This finding raises concerns about the potential for horizontal gene transfer of resistance genes. PCR-based sequencing confirmed the presence of *bla*_KPC-2_ in *C. youngae*. In addition, the plasmid carrying *bla*_KPC-2_ was successfully transferred from the *C. youngae* strain to *E. coli* J53 and *E. coli* DH5α. Both conjugants, *C. youngae*-YS01-*E. coli* J53 and *C. youngae*-YS01-*E. coli* DH5α, exhibited resistance to piperacillin–tazobactam, cefoperazone–sulbactam, and aztreonam, with at least a 4-fold increase in MICs for imipenem and a 60-fold increase for meropenem. Notably, *C. youngae*-YS01-*E. coli* DH5α showed higher MIC values for carbapenem antibiotics than *C. youngae*-YS01-*E. coli* J53 (Table 1). The conjugate *C. youngae*-YS01-*E. coli* J53 was resistant to piperacillin–tazobactam and aztreonam but sensitive or with intermediate susceptibility to imipenem and meropenem. In contrast, the conjugate *C. youngae*-YS01-*E. coli* DH5α was resistant to piperacillin–tazobactam, cefoperazone–sulbactam, and aztreonam but showed intermediate susceptibility or resistance to imipenem and meropenem. The MICs of meropenem and imipenem in both conjugates and the transformants increased by at least 4- and 60-fold, and 16- and 120-fold, respectively, compared to the recipient strains *E. coli* J53 and *E. coli* DH5α (Table 1).

### Sequence analysis, clone experiment, and functional study of *bla*_CMY-190_

We conducted phenotypic and molecular characterization as well as a functional study of a new CFE subtype designated *bla*_CMY-190_, which is encoded by the chromosomal ampC gene and produced by clinical isolates of *C. youngae*. Sequencing of *C. youngae*-YS01 revealed an open reading frame (ORF) of 1,143 bp in length, encoding a putative protein of 381 amino acids. This protein showed a high identity (88.05%) with the plasmid-encoded enzyme CFE-1 (accession number NG_048757) when compared with the amino acid sequence of AmpC β-lactamase. The sequence of the cloned DNA fragment and the associated β-lactam drug resistance pattern identified a new CFE-1-like gene distinct from the *E. coli* plasmid genes for the transcriptional regulator AmpR and AmpC β-lactamase. The deduced amino acid sequence confirmed that the new gene encoding the β-lactamase CMY-190 (Sequence Information: OR896917) is a variant of CFE-1, characterized by 26 amino acid substitutions. In particular, *bla*_CMY-190_ confers resistance to third-generation cephalosporins, including ceftriaxone, cefotaxime, and ceftazidime. Compared to the recipient *E. coli* DH5α-PHSG398, the MICs of cefotaxime, ceftriaxone, and ceftazidime increased 4-fold, while the MICs of cefepime and cefoxitin increased 8- and 32-fold, respectively (Table 2). To assess the inducibility of *bla*_CMY-190_ expression, we measured β-lactamase activity in *E. coli* DH5α strains carrying plasmid pHSG398 using the spectrophotometric method with the chromogenic β-lactam (cephalosporin) nitro-cephalosporin as a substrate. After exposure to a β-lactam inducer (cefoxitin 10 μg/mL or cefotaxime 8 μg/mL), the β-lactamase activity in the bacterial cells was evaluated. The results showed limited induction of β-lactamase activity after exposure to cefotaxime, whereas cefoxitin did not induce further β-lactamase activity. In a parallel experiment, *S. marcescens* was used as a positive control. When exposed to cefotaxime (8 μg/mL), β-lactamase activity increased 4-fold. In contrast, *E. coli* DH5α transformants lacking the *AmpR* plasmid exhibited lower β-lactamase activity and MICs than those carrying *AmpR*, indicating that the expression of the *bla*_CMY-190_ gene requires a functional *AmpR* regulator.

**Table 2:** *In vitro* susceptibility of *C. youngae*-YS01*, C. youngae*-YS01*-E. coli* DH5*α* clone strain, and the *E. coli* DH5α-PHSG398.

Strain	β-lactamase	MIC (μg/mL)														
		IPM	MEM	MEV	CZA	AMK	FEP	CAZ	CRO	CTX	CIP	SCF	FOX	TGC	COL	SXT
*C. youngae*-YS01	CMY-190, KPC-2, CTX-M-3	8	16	0.06	1	2	32	32	32	32	8	64	64	0.125	0.25	16
*C. youngae*-YS01-*E. coli* DH5α	CMY-190,	0.125	0.03	0.03	0.125	2	2	1	1	1	0.06	1	8	0.125	0.25	0.25
*E. coli DH5α-PHSG398*	-	0.125	0.03	0.03	0.125	2	0.25	0.25	0.25	1	0.06	1	0.25	0.125	0.25	0.25

IPM, imipenem; MEM, meropenem; MEV, meropenem–vaborbactam; CZA, ceftazidime–avibactam; AMK, amikacin; FEP, cefepime; CAZ, ceftazidime; CRO, **c**eftriaxone; CTX, Cefotaxime; CIP, ciprofloxacin; SCF, cefoperazone–sulbactam; FOX, cefoxitin; TGC, tigecycline; COL, colistin; SXT, trimethoprim–sulfamethoxazole.

### Analysis of the plasmid and genetic environment of the *bla*_KPC-2_ and *bla*_CMY-190_ genes

**Figure 1:**
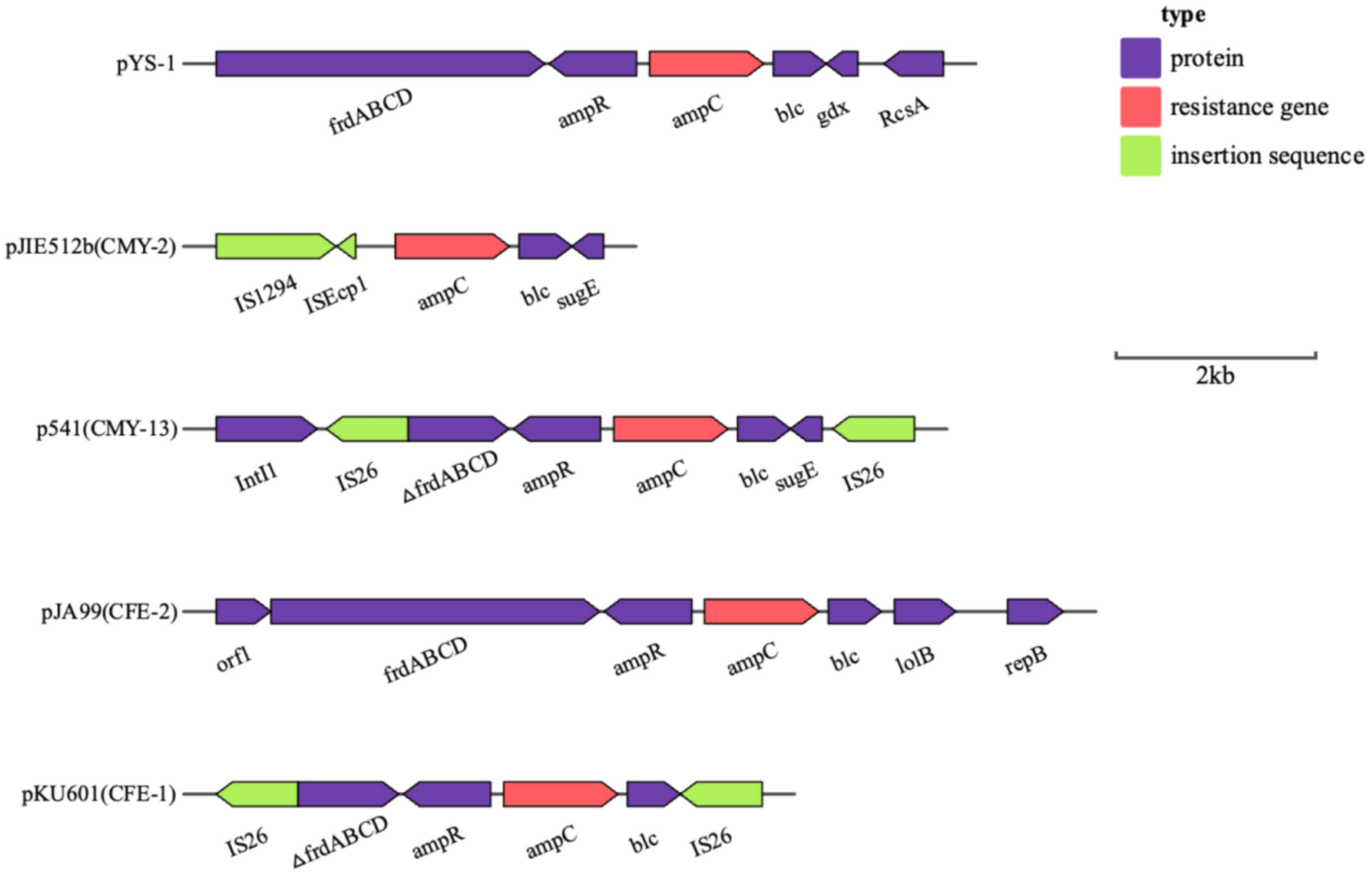
Schematic representation of the genetic background of the *bla*_CMY-190_ gene in *C. youngae*-YS01. Gene and intergenic region sizes are plotted to scale. The open reading frame is shown, with the direction of transcription indicated by thick arrows.

**Figure 2:**
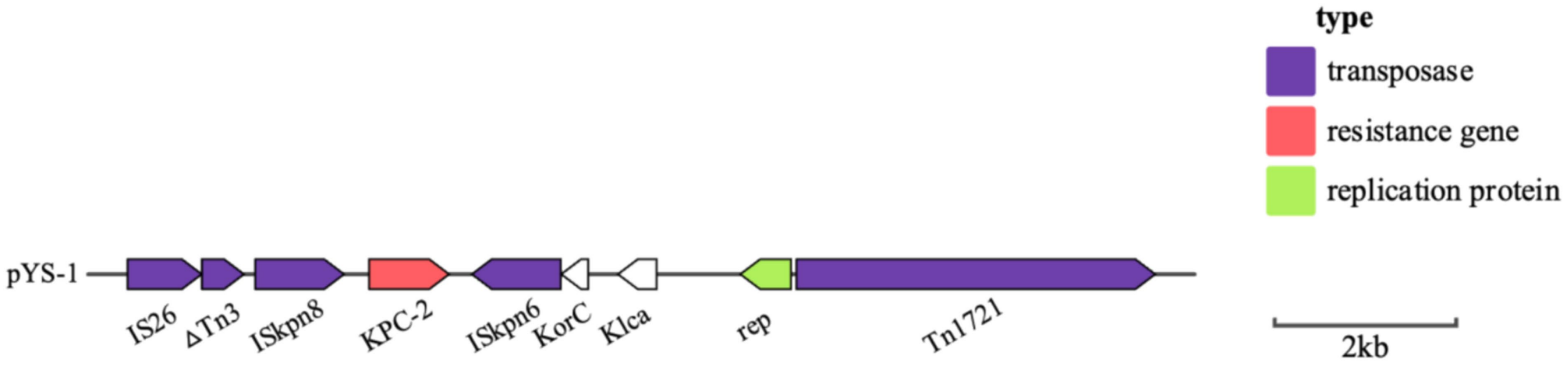
Schematic representation of the genetic background of the *bla*_KPC_ gene in *C. youngae*-YS01. Gene and intergenic region sizes are plotted to scale. The open reading frame is shown, with the direction of transcription indicated by thick arrows.

**Figure 3:**
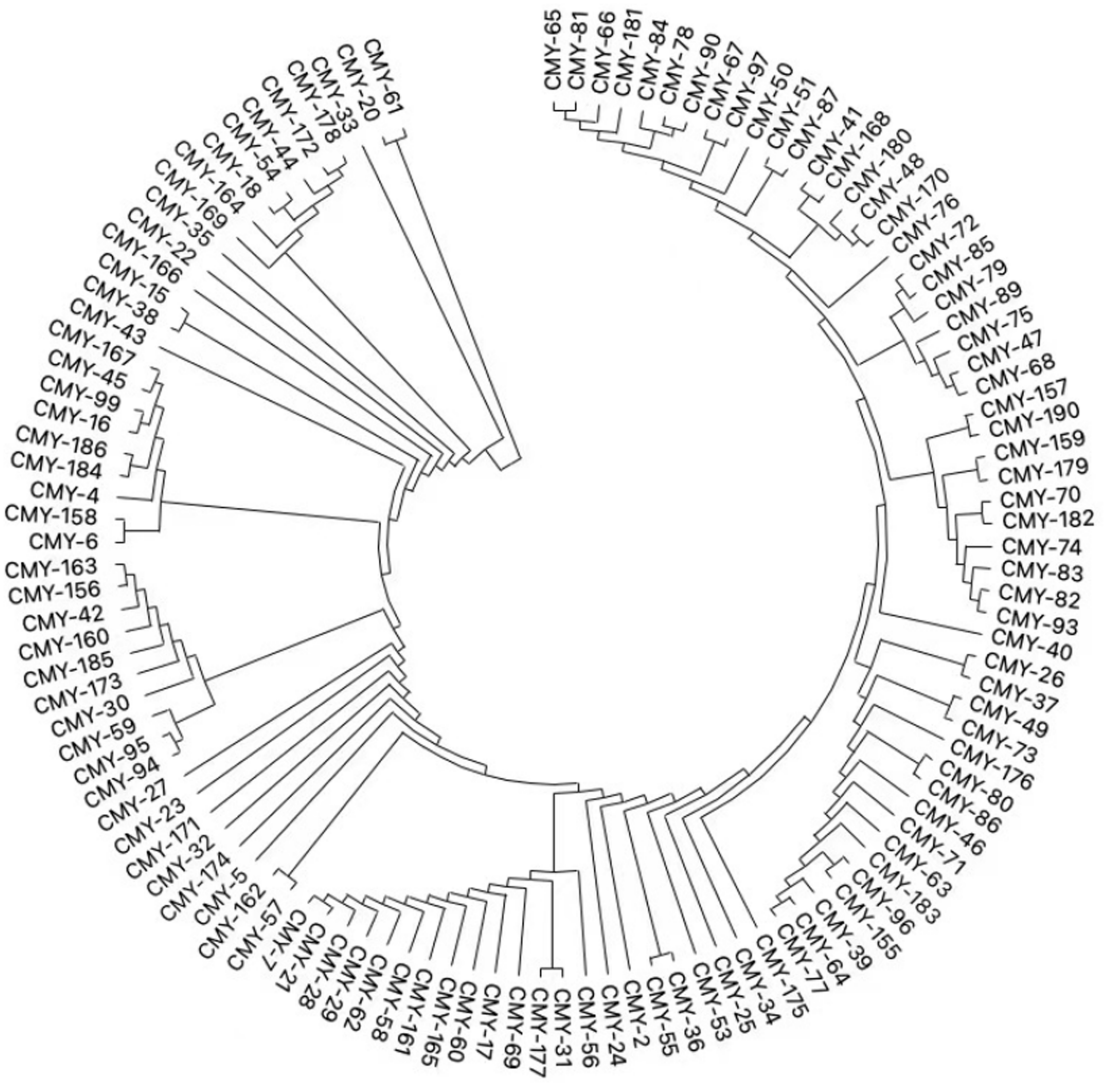
Phylogenetic tree of relative *bla*_CMY_ variants.

**Figure 4:**
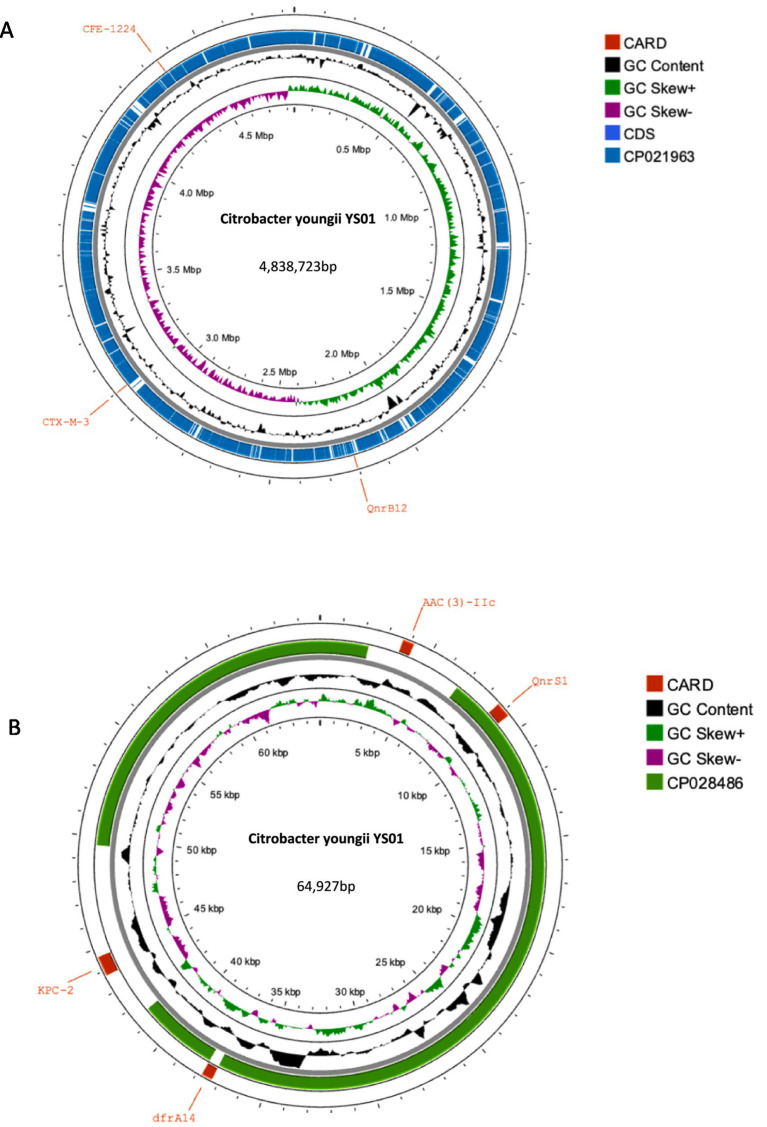
**(A)** Alignments of chromosomes. Comparison of the chromosomes *C. youngae*-YS01 and L6 (CP021963) using Proksee. **(A)** BLAST search for the sequence in GenBank showed that the sequence of *C. youngae*-YS01 was very similar (99.95% coverage and 100% identity) to that of L6 (4,945,156 bp, GenBank accession no. CP021963), a chromosome of *C. youngae* isolated from Korea. **(B)** Alignments of plasmids. Comparison of the plasmids pYS-1 and E41-1 (CP028486) using Proksee. A BLAST search for the sequence in GenBank showed that the sequence of *C. youngae*-YS01 was very similar (79% coverage and 99.92% identity) to that of E41-1 (52,864 bp, GenBank accession no. CP028486), a plasmid of *E. coli* isolated from China.

Whole genome sequencing (WGS) has identified several resistance genes, including the β-lactamase genes *bla*_CMY-190_, *bla*_KPC-2_, and *bla*_CTX-M-3_; the aminoglycoside resistance gene *aac (3)-II*; and the fluoroquinolone resistance gene *qnrS1*. Together, these genes confer resistance to carbapenems, aminoglycosides, and quinolones. Restriction maps and nucleotide sequences were generated to further characterize these elements. Analysis of the *bla*_CMY-190_ sequence revealed the presence of the genes *blc* (encoding an outer membrane lipoprotein), *RcsA* (encoding a transcriptional regulatory protein), and *epmB* (L-lysine 2, 3-aminomutase) downstream of *bla*_CMY-190_. In addition, the *ampR* gene and the *frdABCD* operon of *C. youngae* were identified upstream of the *ampC* gene. The region surrounding the *ampR*–*ampC* genes in *C. youngae* contained both *blc* and *frdABCD*, suggesting a likely common chromosomal location. This genetic element appears to be associated with all known *bla*_CMY-190_-carrying structures, whether chromosomal or plasmid-based (Figure 1). The *bla*_KPC-2_ gene was found to be flanked by mobile elements related to Tn*1721* and IS*26*, consistent with the predominant genetic structures carrying *bla*_KPC-2_ in the domestic environment (Figure 2). This genetic configuration has recently been identified in several *Citrobacter* species (Zhu et al., 2020; Qiao et al., 2023; Hu et al., 2019). Sequencing results from *C. youngae* revealed that the *CFE* gene is located on chromosome 4878723bbp within the ST195 sequence type. Other chromosomal resistance genes include *bla*_CTX-M-3_ and *QnrB12*. Comparative BLAST analysis indicated high similarity between this *C. youngae* chromosome sequence and a previously isolated strain from Korea (GenBank accession no. CP021963). A phylogenetic tree illustrating the related *bla*_CMY_ variants is shown in Figure 3. The *bla*_KPC_ gene was identified on a 64,927 bp IncN plasmid, which, based on BLAST analysis, shows high similarity to the plasmid sequence of an *E. coli* strain isolated from a hospital in Shanghai, China (79% coverage, 99.92% identity, GenBank accession no. CP028486) (Figure 4).

## Discussion

AmpC-type β-lactamases are classified as class C enzymes, with different expression patterns depending on their genetic context. These include the inducible expression of chromosomal AmpC β-lactamase, stable de-expression with inducible expression, and plasmid-mediated AmpC β-lactamase. Among these, the *bla*_CMY-2_ gene is the most widely reported AmpC gene worldwide. *bla*_CMY-2_, initially identified in *E. coli* and *Salmonella* isolates, is often associated with transposons, facilitating its transmission across community and healthcare settings. The ability of plasmid-mediated *bla*_CMY-2_ to transfer between bacterial species has contributed to its global prevalence, with the incidence in Egypt increasing from 2.91% in 2019 to 4.09% in 2020 and in the United States increasing from 1.3% in 2016 to 3.42% in 2019 (Rodríguez-Guerrero et al., 2022). Typically, the *bla*_CMY_ gene is associated with the IS*Ecp1* element, forming the IS*Ecp1 tnpA-bla*_CMY-2_-*blc*-*sugE* arrangement, largely due to a strong promoter within IS*Ecp1* (Chiu et al., 2021). This genetic configuration, in combination with host factors, significantly influences ceftriaxone susceptibility. Under increasing selective pressure, *bla*_CMY_ variants with hydrolytic activity against broad-spectrum cephalosporins have emerged, conferring resistance to agents such as cephalosporins, cefoxitin, and aztreonam and even reducing susceptibility to fourth-generation cephalosporins (e.g., cefepime). In addition, the *bla*_CMY_ gene, in combination with altered membrane permeability, may contribute to imipenem resistance in *K. pneumoniae*. Of particular concern, *bla*_CMY_ variants also reduce the susceptibility of *Enterobacterales* to avibactam. For example, Tyr150Ser and Asn346Ile substitutions in CMY-2 increase resistance to avibactam. In addition, *bla*_CMY-172_ and *bla*_CMY-178_ are associated with ceftazidime–avibactam resistance in Enterobacterales, with CMY-178 showing a higher level of resistance than CMY-172 (Merida-Vieyra et al., 2020; Zhou et al., 2023).

In this study, we identified a novel AmpC β-lactamase gene, *bla*_CMY-190_, in clinical isolates of *C. youngae* in China. This is the report of a chromosomally encoded AmpC β-lactamase gene, *bla*_CMY-190_, carrying the regulatory *ampR* gene derived from the *C. youngae* plasmid. Regulation of AmpC β-lactamase expression is closely linked to cell wall recycling and involves the *ampR* gene, which encodes a transcriptional regulator from the *LysR* family (Balasubramanian et al., 2014). Research suggests that *AmpR* binds to a 38-base pair sequence within the intergenic region between *ampR* and *ampC* genes. In the absence of β-lactam inducers, *AmpR* downregulates β-lactamase synthesis by 2.5-fold, whereas in their presence, expression can be increased by 10- to 200-fold (Nakano et al., 2004). Our results describe the sequence structure surrounding the *ampR*–*ampC* regions, including *bla*_CMY-190_. Unlike most plasmid-encoded AmpC β-lactamase genes, such as *bla*_CMY-2_, *bla*_CMY-4_, and *bla*_LAT-1_, which lack the *ampR* gene (Biez et al., 2023), *Citrobacter* spp. *and Enterobacter* spp. have complete *ampR* and *ampC* genes. In addition, these species have a downstream fumarate operon (*frdABCD*) adjacent to the *ampR* gene and an outer membrane lipoprotein (*blc*) located downstream of the *ampC* gene (Nakano et al., 2004). Analysis of the *bla*_CMY-190_ gene reveals a close relationship with the chromosomally encoded AmpC β-lactamase gene in *C. freundii.* The amino acid sequence of *bla*_CMY-190_ shares 88.05% identity with that of *CFE-1* from *C. freundii* JA99, which was isolated from clinical samples in China (Chen et al., 2018). In addition, the amino acid sequence of *AmpR* shares 99.0% identity with the *AmpR* sequence of *C. freundii* JA99. This high degree of similarity strongly suggests that the *bla*_CMY-190_ gene is derived from the plasmid *ampC* gene of *C. freundii* JA99. This hypothesis is further supported by the presence of the *ampR* and *ampC* genes, as well as the *frdABCD* operon and the *blc* gene, which surround the *ampR* and *ampC* genes in *C. freundii* species.

Based on previous epidemiological studies, KPC-type carbapenemases are more common in CRE, while their identification in *Citrobacter* spp. is relatively rare. In *Citrobacter* spp., OXA-48-like carbapenemases are the predominant type, followed by NDM and VIM variants (Biez et al., 2023). Notably, while KPC-type carbapenemases are highly prevalent in CRE overall, they are less frequently detected in *Citrobacter* spp. Our study identified a strain of *C. youngae* carrying the *bla*_KPC-2_ gene, making it the first detection of this gene in *C. youngae*. As a clinically important opportunistic pathogen, *C. youngae* poses a significant public health challenge due to its carbapenem resistance. This case illustrates how *C. youngae* can act as a silent reservoir for critical resistance genes. The emergence of the *bla*_KPC-2_ gene in *C. youngae* deserves widespread attention due to its potential impact on clinical infection management. Genetic elements play a crucial role in the spread of resistance genes. In many countries and regions, including Europe (Naas et al., 2008), the United States (Chen et al., 2013), and Brazil (Pereira et al., 2013), the *bla*_KPC_ gene is mainly located on mobile elements such as Tn*4401* and Tn*3*-Tn*4401*. These composite transposons belong to the Tn*3* family and contain *bla*_KPC-2_, transposase, resolvase, and insertion sequences IS*Kpn6* and IS*Kpn7*, which allow high transposition frequencies for *bla*_KPC-2_. In Asia, *bla*_KPC-2_ is mainly found on different variants of Tn*1721* and IS*26* (Fu et al., 2019). Studies indicate that the KPC-2-carrying plasmid in *C. youngae* is closely related to plasmids isolated from *K. pneumoniae* in Suzhou. These plasmids share a composite transposition element consisting of Tn*1721* and IS*26*. Homologous recombination mediated by IS*26* is likely to lead to the rearrangement and widespread dissemination of these resistance regions, suggesting a potential transfer of *bla*_KPC-2_ from *K. pneumoniae* to *C. youngae*. The transferability of the plasmid was further confirmed in conjugation experiments. Compared to other *Enterobacterales*, *C. youngae* poses a significant risk for the transfer of antimicrobial resistance, which becomes an additional concern factor in clinical settings (Nüesch-Inderbinen et al., 2013; Hawkey et al., 2001). Therefore, early identification of resistance mechanisms and resistance gene transfer pathways is essential to improve clinical anti-infection strategies and to control the potential widespread of *Citrobacter* spp.

## Conclusion

This study reports the first identification of a *C. youngae* strain carrying both the chromosomally encoded AmpC β-lactamase gene *bla*_CMY-190_ and the plasmid-encoded carbapenemase *bla*_KPC-2_, detected in a patient’s ascites sample. The patient presented with multiple complications, including hepatic malignancy, decompensated cirrhosis due to hepatitis B, ascites, acute renal failure, uremia, and portal vein thrombosis, and had been treated with a range of antibiotics. The newly identified cAmpC β-lactamase (CMY-190) showed activity against oxyimino-cephalosporins. Our findings highlight the need for increased surveillance and prevention efforts targeting CMY-190 to reduce the spread of multidrug-resistant Gram-negative bacilli in healthcare settings. The detection of *bla*_KPC-2_ in *C. youngae*, possibly by horizontal transfer from *K. pneumoniae*, is also a first and highlights that *C. youngae* and other less-reported *Enterobacterales* may serve as unrecognized reservoirs for carbapenemase genes.

## Data Availability

The datasets presented in this study can be found in online repositories. The names of the repository/repositories and accession number(s) can be found at: Sequence Information: OR896917.

## Footnotes

^1^ https://github.com/tseemann/mlst

^2^ https://proksee.ca/
